# Abstracts and Gleanings

**Published:** 1883-08-20

**Authors:** 


### ABSTRACTS AND GLEANINGS.

**Practical Medicine.**—At the American Medical Association Dr. J. H. Hollister, of Illinois, Chairman of the section on Practical Medicine, read his address. He declared that progress in medicine depended on the exercise of pure reason, which can never defer to faith, and can expect no such aid from revelation as may cause it to walk with a wisdom not its own. It is so much the creature of mental and material forces which are interchangeable, interdependent and inseparable, that reason is compelled to thread her way with steps slow and uncertain, sometimes in truth, ofttimes in error, ever painfully conscious of her weakness and of the mysteries which confront on every side. The absence of full positive knowledge has prevented the restraint of the fancies and credulities within its fold, and, as a consequence, speculations have been piled mountain high by one generation but to disappear in another like chaff before the driving wind; but there have been left after these thorough winnowings some golden truths, and the treasure-house is being slowly, but surely enriched by the garnerings of the ages.

He complimented the profession on the growing number of original workers, and on the consequent improved quality of the literature of the profession. He declared that, probably, no year in the history of medicine had witnessed as much original work as had the year just ended, or seen a better periodical literature.

In referring to the labors of Koch, he maintained that, while the tubercle bacillus undoubtedly existed, the question whether it was an effect or a cause of tuberculosis still remained unsettled, and that it will continue to so remain until a means is discovered of destroying it without injury to the tissues.

He called on the profession to take some action looking toward a restoration of the evils which attend the sale of proprietary medicines, and suggested a somewhat doctrinaire method of ridding the country of this curse.

He touched pointedly on the evils of a combination of the teacher and examining power in our colleges, and suggested the creation of a central bureau, to whom should be relegated the examining and licensing power.

The address was, in the main, pithy and practical.

Dr. J. K. Bartlett, of Wisconsin, Chairman of the Section on Obstetrics and Diseases of Women, not being in attendance, his address was read for him by Dr. Senn, of Milwaukee. He criticized Emmett's operation as a routine procedure, stating that experience has shown that undue importance has been attached to the lesion which it is designed to overcome, and that it fails to uniformly work the relief contemplated. A more definite understanding of the conditions requiring its performance is demanded.

Of Battey's operation, he said that most of the indications which have been claimed to warrant it are now generally admitted. There is still some diversity of opinion concerning its value in epilepsy, hystero-epilepsy and mania, seemingly dependent on, or associated with ovarian troubles.

He discussed the employment of electricity as a means of destroying the fœtus in extra-uterine pregnancy, and illustrated its value by the report of several cases.

In regard to transfusion in post-partum hæmorrhage, he had concluded that, while it is theoretically valuable it is practically unsatisfactory. He related cases in which milk and saline solutions had been employed instead of blood, with benefit.

On the whole, he believed that more harm than good was being done by tne use of the obstetric forceps. He referred, with keen sarcasm, to the ingenuity of man in devising mechanical contrivances for the emptying of the human uterus, and would not be surprised at any time to hear of the introduction of a one-horse electro-motor power, so arranged as to do the pulling, and thus save the obstetrician, as well as the mother, any exertion.

He gave his own experience in the use of ergot in labor. He had employed it for many years where the second stage was retarded by insufficient contractions and where no pelvic obstacle existed, and he regards it as a valuable resource and devoid of danger in such cases.

His opinion of anæsthetics in labor may be inferred from the statement witn which he concluded his reference to the subject. If an anæsthetic ever produced post-partum hemorrhage, injury to the child, or other than beneficial results, experience tells us it must be due to the impurity of the anæsthetic employed, or to want of that experience and discretion which is necessary, not only here, but in the use of all therapeutic measures employed for the relief of human suffering.

Dr. Bartlett concluded his eminently able paper with a plea for greater attention to constitutional treatment in pelvic disease thau is fashionable in these days of rampant specialism. "No one," he said, "who has not received thorough training in general medicine and has not tested, confirmed and enlarged his natural thought through many years of general practice, is fitted for a specialist. When medical gynecology is thus studied and practiced, aided by larger general, local, therapeutic and hygienic rdsources, which such research in time will develop, tha clearer and surer will be the diagnosis which the future will bring, and the time will come when the present brilliant triumphs of the surgical gynecologist will grow pale before the achievements of his medical co-worker."

**Deaths During the Administration of Anæsthetics.**—Mr. J. H. Lee MacIntire, Medical Superintendent of the Bristol Royal Infirmary, reports the following cases in the British Medical Journal:

A man, aged 54 years, was admitted to the Bristol Royal Infirmary December 30, 1881, suffering from a strangulated inguinal hernia of sixty-four hours standing. He had vomited almost incessantly from the first, and for the last twelve hours the vomited matter had been fæcal. On admission, his tongue was moist, his pulse weak but regular, and his aspect somewhat pinched. Chloroform was administered preparatory to an attempt at reduction by taxis, and everything went on well for the first minute and a half, a little over one drachm being inhaled, and this amount was divided into three parts. He then commenced to struggle a little, and his pulse was noticed to have improved, when he was seen to be about to vomit. The vomited matter measured almost a pint, and was stercoraceous and very fluid. Loud tracheal rales were now heard and the breathing for the first time became embarrassed. He was immediately turned over, when nearly two quarts of fluid were ejected. His pupils were now widely dilated. Artificial respiration, inversion, cold affusion, and dragging forward of the tongue, were at once tried ; air entered the lungs freely, there were no tracheal rales, and the pupils became contracted. He now vomited again, or, rather, some more fluid poured out of his mouth. Attempts to resuscitate him were persisted in for over twenty minutes, but without avail. From the first arrest of pulse and respiration, neither heart-beat nor voluntary attempt at respiration was noticed. The first vomit occupied about a minute.

The post-mortem examination showed the heart healthy, aorta slightly atheromatous, kidneys granular, and a small quantity of food, which appeared to be partly digested milk, and which was about as large of a pea, was lodged just below the rima glottidis.

A woman, aged 45 years, who had been in the ward some days with an abdominal tumor, was, on April 19, 1883. examined under the influence of an anæsthetic mixture consisting of one part of chloroform to three parts of ether. She was known to have chronic bronchitis, and was suspected of phthisis at the right apex. She had taken some beef-tea and egg a short time before examination. She took the anæsthetic very well, becoming unconscious in three minutes, and remaining so for ten, when her breathing was noticed to be growing shallow, but her pulse, color and pupils remained unaltered. She took three respirations, each more shallow than its predecessor, and gave signs of being about to vomit. She was just about to be turned over on her left side, when her breathing stopped, whilst her heart could still be seen acting. Her pulse then failed, her face became livid, and her pupils about two-thirds dilated. Inversion and artificial respiration were immediately tried, and air entered the lungs freely, with a total absence of tracheal rales. The pupils were now noticed to be about three-fourths dilated, and some half-digested liquid food oozed out of her mouth. In case any might have entered the larynx, although there was no reason to suspect such an accident, tracheotomy was performed. Artificial respiration was kept up for half an hour, and inhalations of nitrite of amyl, injections of ether, cold affusion, and an enema of brandy were also unsuccessfully tried, the patient showing no sign of returning animation from the first, with the exception of closing her jaws firmly about five minutes after the commencement of artificial respiration.

Post-mortem examination showed the heart-vessels and brain to be healthy, and there was no food in the air-passages. The abdominal tumor was due to tubercular peritonitis, and there was general bronchitis, and some tubercle was found in the apex of the right lung.

In both cases the anæsthetic was administered on a flannel mask which covered the nose and mouth.

A third case was that of an elderly man in the Western Infirmary, Glasgow, and the anæsthetic used was chloroform. The patient was to undergo the operation of excision of the tongue. The anæsthetic was given, as is usual in the Western Infirmary, by means of a towel, and it had not been very long administered before it was noticed that respiration had ceased, that there was marked lividity of the face, and that the patient presented many ot the features of a person in the tonic stage of an epileptiform convulsion. Every effort was made to re-establish breathing, but without success.

A post-mortem examination revealed nothing to account for death, beyond the presence of well-marked symptoms of asphyxia. This is the third death that has occurred in the Western Infirmary during the administration of chloroform, and its occurrence just at the present time is a atriking comment on the chloroform agitation recently carried on at the Royal Infirmary; for in this case the anæsthetic was administered by the surgeon himself, with all the care that extensive knowledge and experience of the subject could give.—*Phila. Med. Times.*

**Successful Laparotomy for Intestinal Obstruction—Listerism without Spray.**—A woman, 53 years of age, previously in good health, under the care of Mr. Alder Smith, was seized with acute pain just above the umbilicus, which was severe and paroxysmal, simulating biliary colic. The abdomen was normal, without tenderness or swelling. There was constipation, vomiting of bile-stained fluid, and evident obstruction of the intestine. Morphia was given in quantities sufficient to control the pain, and several doses of castor oil were administered, but were vomited in the course of an hour afterwards. A large enema of four pints of warm sweet oil, given through a stomach-tube, introdued into the colon, did not relieve the obstruction, although it brought away a few pieces of hard fæcal material. No tumor could be detected through the abdominal wall. The abdomen became swollen by tympantic distention of the ileum, and the patient had the appearance of one suffering with strangulated hernia. The operation of laparotomy was decided upon after consultation, and the patient's consent obtained. She was at this time, on the fourth day after the first appearance of the symptoms, deeply under the influence of morphia, temperature 98.6°, pulse 90, belly typanitic. She had been vomiting very slightly, no nourishment having been given except a little beef-esence and ice-water. The apartment was kept at a temperature of 95°, and the air moistened with steam. All the instruments were thoroughly washed in carbolic lotion (1;40), and afterwards dried; the operator's hands and the patient's abdomen were likewise washed and dried. The operation was done by Mr. Savory. No carbolic spray was used, and no carbolic lotion or other irritant was allowed to enter the peritoneal cavity.

The abdomen was opened by an incision extending from the umbilicus to the pubes; the intestines were much distended with flatus. Tracing them downwards, a portion of the ileum was found tightly nipped by some band or constriction; below this the intestine was flaccid and empty. On pulling this portion of the intestine and breaking through a band, a loop of strangulated intestine came out; this was intensely congested, of a deep claret color, and approaching a condition of gangrene As soon as the constricted portion was found to be pervious to flatus, no unnecessary exploration was made as to the exact cause of the constriction, but the intestines were at once returned. A little blood-stained fluid was removed by new and clean sponges from the abdomen, and the wound was closed by silk and silver sutures; there was very little bleeding, and no vessels had to be tied. The dressing consisted of lint soaked in carbolized oil (1 in 40), long strips of plaster, and a broad flannel bandage. Morphia (gr. 1/3) was given as soon as the patient recovered from the chloroform. Ice-water only was ordered, and five minims of the tincture of opium every hour, the temperature of the room to be maintained at 95°. The dressing was re-applied on the third day, when the wound was found to have almost entirely healed by first intention, no pus, no peritonitis. The greatest care was observed with regard to diet; she had only a small amount of essence of beef, besides the ice-water, for a week after the operation. On the eighth day she was permitted to take some bread and milk and custard-pudding. She made a prompt and perfect recovery.—*British Med. Jour., May 26.*

**Extraction of Deciduous Teeth.**—The order of shedding and of eruption shows that the first to change places are the central incisors; secondly, the lateral incisors; thirdly, *not* the canines, but frequently the second molar for the second bicuspid; fourthly, the remaining molar for the other bicuspid, and lastly the canines.

In a normal condition the jaw bones continue their growth after the growth of process about the temporary teeth has ceased, and thus, as the period approaches for the eruption of the permanent teeth, we find spaces between the temporary ones, thus enlarging the alveolar arch for the accommodation of the larger members of the permanent group.

The growth or enlargement of that part of the jaw upon which the deciduous dental arch is situated seems to have obtained its complete development at the period of shedding, and the incisors and bicuspids will find room equal to their necessities.

The premature extraction of any or all even of these eight named teeth will not interfere with the natural and expected enlargement of the jaw, but the premature extraction of the canine teeth will be likely to lead to most serious results.

After the jaw bone has ceased its enlargement there seems an almost universal tendency for the bicuspids and molars to crowd to the anterior part of the mouth, and to fill any space in the alveolar arch that may not already be occupied. This is not only true in the formative period, but is equally true in adult life, if the occlusion of the opposing jaw does not conteract it. The consequence of this inevitable tendency is, that unless the temporary canines remain in their places until their permanent successors are ready to emerge, the bicuspids and whatever molars are behind them will crowd forward and occupy the space which belongs to the canines.

We thus see that, whatever may be the inducement to remove any or all of the deciduous teeth prior to their period of shedding, the canines should be retained until there is ample evidence of the early emergence of their permanent successors, unless the health and comfort of the child would be sacrificed in so doing. But it would be far better to remove one or all of the deciduous teeth and take the risks of irregularity in the permanent ones, than to submit the child to constant suffering and consequent injury to its health by their retention.

In a case of retarded dentition the writer takes issue with the practitioner who removes the deciduous teeth when the usual period arrives for shedding, regardless of any evidence that the permanent ones are ready to erupt. His reason is, that the retention of the temporary tooth retards the growth of the permanent one.

In this issue is involved the function of absorbtion, and his practice would indicate that the non-absorption of the temporary tooth was the primal cause of the retarded dentition, rather than that retarded dentition is the cause of the non-absorption. Cause and effect are, in his mind, evidently transposed.

Such a practice will unquestionably lead, in many cases, to serious results.

It is not always certain, when there is no outward indication, that a tooth lies concealed.

It is not a very uncommon thing to see some one tooth of the permanent set missing, and to learn that it never erupted. And again, a retarded dentition generally indicates teeth of better organization and less liable to decay than those which have developed at an earlier age.

So long as deciduous teeth remain in the jaw in a firm and undecayed condition, with no evidence of a misdirection of their permanent successors, it is not advisable to remove them.—*Independent Practitioner.*

**Calomel in Croup—Colchicum in Gout.**—Dr. Jno. H. Trent, of Brooklyn, N. Y., favors us with the following brief extracts from the life of the famous Sidney Smith:

"A few months after the birth of his daughter, he went to the sea-side for his health, where, but for his courage and firmness, he would have lost her in a way he had not anticipated. When only six months old, she fell ill of the croup, with such volume that it defied all the remedies employed by the best medical men there. The danger increased with every hour. Dr. Hamilton, one of the most eminent medical men in Edinburgh, was sent for. He could not come, but said: 'Persevere in giving two grains of calomel every hour; I never knew it to fail.' It was given for eleven hours. The child grew worse and worse, The medical man in attendance then said, 'I dare give no more; the child must die. I can do no more, but at this age I would not venture to give more calomel to my own child.' 'You said you can do no more. Hamilton says, 'Persevere.' I will take the responsibility. I will give it to her myself.' He gave it, and the child was saved.

" 'I am suffering from my old complaint, the 'hay-fever,' as it is called. My fear is perishing by deliquescence. I melt away in nasal and lachrymal profluvia. My remedies are warm pediluvia, cathartics, topical application of a watery solution of opium to eyes, ears and the interior of the nostrils. The membrane is so irritated that light, dust, contradiction, an absurd remark, the sight of a dissenter—anything, sets me sneezing; and if I begin sneezing at 12, I don't leave off till 2 o'clock, and I am heard distinctly in Taunton, when the wind sets that way, a distance of six miles. Turn your mind to this little curse. If consumption is too powerful for physicians, at least they should not suffer themselves to be outwitted by such little upstart disorders as the hay fever.'

"On observing some autumn crocus in flower, he stopped. 'There,' he said, 'who would guess the virtue of that little plant? But I find the power of colchicum so great that if I feel a little gout coming on, I go into the garden and hold out my toe to that plant, and it gets well directly. I never do more without orders from headquarters."

**Don't.**—Don't go to bed with cold feet. Don't sleep in the same undergarments that are worn during the day. Don't sleep in a room that is not well ventilated. Don't sit or sleep in a draught. Don't lie on the left side too much. Don't lie on the back, to keep from snoring. Don't try to get along with less than seven or eight hours' sleep out of twenty-four. Don't jump out of bed *immediately* on awaking in the morning. Don't forget to rub yourself well all over with crash towel or hands before dressing. Don't forget to take a good drink of pure water before breakfast. Don't take long walks when the stomach is entirely empty. Don't start to do a day's work without eating a good breakfast. Don't eat anything but well-cooked and nutritious foods. Don't eat what you don't want just to save it. Don't eat between meals, nor enough to cause uneasiness at meal time. Don't eat the smallest morsel unless hungry, if well. Don't try to keep up on coffee or alcoholic stimulants, when nature is calling you to sleep. Don't stand over hot-air registers. Don't inhale hot air or fumes of any acids. Don't fill the gash with soot, sugar, or anything else to arrest the hemorrhage when you cut yourself, but bring the parts together with strips of adhesive plaster. Don't wear thin hose or light-soled shoes in cold or wet weather. Don't strain your eyes by reading on an empty stomach or when ill. Don't ruin your eyes by reading or sewing at dusk, by a dim light or flickering candle, or when very tired. Don't sing or hollow when your throat is sore or you are hoarse. Don't drink ice water when you are very warm, and never a glassful at a time, but simply sip it slowly. Don't take some other person's medicine because you are similarly afficted. Don't bathe in less than two hours after eating. Don't eat in less than two hours after bathing. Don't call so frequently on your sick friend as to make your company and conversation a bore. Don't make a practice of relating scandal, or stories calculated to depress the spirits of the sick. Don't forget to cheer and gently amuse invalids when visiting them. Don't call on your sick friend and advise him to take some other medicine, get another doctor, eat more, eat less, sit up longer, go out more frequently, stay a week, or talk him to death, before you think of leaving.—*Scientific Californian.*

**Treatment of Nasal Catarrh.**—To this we might add chronic pharyngitis, which, possibly, may extend into the larynx and trachia. As Buffalo is the *hub* around which all catarrhs move, and as cases without number are calling every week seeking relief from this unpleasant and frequently destroying disease, all the resources of the physician is brought into action to cope with it. The writer has used everything that he has ever read or heard of in the shape of medication, douches, inhalations, etc., but has never met with sufficient success to be enabled to guarantee a cure. Indeed the results were so unsatisfactory that the hope of success in curing catarrh had almost disappeared, when the thought presented itself that, possibly, some more active medicines might be applied locally, which would heal the ulcerated surfaces. A curved sponge holder was brought into play with a little absorbent cotton, and a preparation of carbolic acid, ʒ ss; sulphate hydrastia, gr. xv; glycerine, ℥ ss; aqua, ℥ iss; mix; was applied behind the velum as far as the holder could reach, and thoroughly on all sides and everywhere where there was inflammation or ulceration. The tronsformation was wonderful, and cases seemed to recover after six or eight treatments which, before, seemed almost hopeless. The writer has treated about seventy cases in this manner already, and all have either recovered or are on the road to recovery. This may not be original, but it seems far better than long medications. The treatment is very disagreeable, and in some cases quite painful, but if the application is made with the expiration of the breath there is but little danger of strangling, and the smarting may be only momentary or possibly last for four or five hours. It will be noticed, however, that each succeeding treatment will be attended with less pain and discomfort.—*Investigator.*

**Case of Stramonium Poisoning.**—Dr. C. F. Bevan reports the following case in the Medical Chronicle: A boy, æt. 12, ate three-fourths of a one-ounce package of the dried leaves, after which he appeared dull and fell asleep. An hour later he was extremely drowsy, pulse 125, temperature 101° F. in axilla, and surface of a uniform bright red from head to foot. Lips, tongue and fauces very dry and swallowing very difficult, the constriction and spasm of the muscles of deglutition suggestive of hydrophobia. Pupils widely dilated and insensible to light; vision defective and inaccurate, everything appearing green. There were delusions of hearing and other cerebral disturbances, accompanied by boisterous laughter. Speech incoherent, intellect impaired, general sensibility greatly blunted. When recumbent the arms were thrown around wildly; when he attempted to walk his movements were like those of a person under the influence of alcohol. When he remained quiet there was subsultus tendinum. Urine high colored and frequently voided. An emetic brought up a large quantity of the weed; the stomach was then thoroughly washed out by means of a stomach pump. Bicarbonate of sodium was given freely, and in two hours three-and-a-fourth grains of sulphate of morphia were administered hypodermically, only slightly diminishing the dilated pupils. He was not allowed to rest until consciousness returned and the redness disappeared. The next day he had no recollection of what had occurred. It was a week before the pupils became normal.—*Maryland Medical Journal.*

**Kairine, a Substitute for Quinine.**—The Medical Press and Circular, June 6th, describes a new drug introdueed to notice in Vienna. It is prepared from chinoline, which is itself obtained synthetically from aniline, nitro-benzol, glycerin and sulphuric acid. The new agent produced from chinoline has been named Kairine, and is technically described as methoxychinolintetrahydride. It is an oil and unites with hydrochloric acid. It has a singular effect on the urine, changing its color to brown or olive green, and sometimes grass green, and also causing the development of bacteria in the urine. It is very active in its effects on the human system. Three grains in twenty-four hours is accounted a large quantity, having produced in one instance a fall of temperature, with shivering and indications of collapse. Prof. Drasche, of Vienna, considers it superior to all other drugs as a prompt anti-pyretic, and believes that it has a great future before it.—*Pacific Med. Jour.*

**A Dentist's Fee.**—A man with too much "sand" in him for professional profit, seated himself in a dentist's chair to try the steel method of curing toothache. He asked the dentist playfully: "I suppose you won't hurt me any?" and the dentist playfully replied: "If I don't hurt you, I won't charge you anything." The countenance of the man of sand immediately assumed an expression resembling a cross between a Quaker and a Stoic; the dentist dexterously applied a forceps having something the appearance of a gridiron struck by lightning, and the decayed basis of that "hell o' oll diseases" was, in an instant, swung aloft. The patient's placid expression never changed; he got down from the chair and remarked on the heavenly beauty of the day:

> "—And wondered whether
> The cloud in the West would bring wet weather,"

adjusted his "tie" and "plug," said adieu, and started away. As he stood in the door, the dentist inquired: "Did I hurt you any?" The reply was, "Not in the least, my dear doctor. I bid you good day." The dentist said, "H'm!"

